# The influence of professionals’ personal views and values in the development of guidelines for rare diseases: an example from phenylketonuria

**DOI:** 10.1186/s13023-025-03983-y

**Published:** 2025-11-10

**Authors:** Annemiek M. J. van Wegberg, Cristina Romani, Francjan J. van Spronsen

**Affiliations:** 1 Division of Metabolic Diseases, Beatrix Children’s Hospital, University Medical Center Groningen, Hanzeplein 1, 9700 RB Groningen, The Netherlands; 2 Psychology Department, Aston University, Birmingham, UK

**Keywords:** PKU, Expert opinion, Preferences, Values

## Abstract

**Background:**

In the process of guideline development, expert opinion is introduced when there is low-quality or no scientific evidence available. During the first revision of the European phenylketonuria guidelines, the limited data regarding adult treatment targets was interpreted and weighted in different ways by different professionals. In this study, we have recorded and analyzed personal experience and views that may affect decisions.

**Methods:**

A web-based questionnaire was sent to all panelists involved in the revision of the guidelines (n = 23), evaluating demographics, opinion about treatment targets, interpretation of scientific data and general views to establish adult specific treatment guidelines.

**Results:**

19 panelists responded. Most acknowledged the importance of maintaining metabolic control even in adulthood with target levels in line with previous recommendations, but that dietary treatment is a limiting factor for social life. 68% preferred the risk of over-treatment rather than undertreatment. The great majority considered cognitive, behavioral and wellbeing measures to be critical to measure treatment success. 58% considered significant group differences of 1 SD or less in neuropsychological tasks to be clinically significant.

**Conclusion:**

Results highlight differences in expert opinions and the importance of making them more transparent to reach less-biased recommendations.

## Background

The term evidence-based medicine (EBM) emerged in 1991, primarily aiming to educate clinicians in assessing and interpreting the results of clinical studies and to integrate these results into their daily practice [1]. EBM and evidence-based practice (EBP) have also been integrated in the development of clinical practice guidelines. Guidelines should be based on the best available evidence. To facilitate this process, several tools and checklists have been developed over the years to appraise the quality of a single study, to assess the total body of evidence available, and to assess the quality of a subsequent clinical recommendation [1]. The grading of recommendations assessment, development and evaluation (GRADE) method is a prominent example. However, it must be acknowledged that evidence alone never determines decisions, it is always evidence in the context of values and preferences [1]. Values and preferences balance the importance of desirable and undesirable outcomes.

It is relatively easy to come to a guideline recommendation when a consistent body of evidence, derived from multiple large and well performed studies, is available [2], but this is an ideal situation which rarely occurs. In rare diseases, the development of evidence-based guidelines is hampered by a lack of high-quality studies with high numbers of participants. When evidence is lacking, is of low-quality or mainly indirect, guidelines often rely on expert opinion. An opinion is a view or judgment formed about something, not necessarily based on evidence [3]. Experts will interpret existing evidence based on their knowledge and experiences: their reading of the literature and their own clinical practice [4]. In these cases, it is important to explore which underlying beliefs and values could play a role in the judgements experts make when formulating such guideline statements.

During the first revision of the European guideline on phenylketonuria (PKU; box 1), the influence of personal views in interpreting evidence was evident. This was especially true when formulating the recommendation on phenylalanine blood levels that adult PKU patients are advised to maintain. Before we reached consensus (defined as ≥ 75% of agreement [5]), this topic was heavily debated.

The same limited data was interpreted and weighted in different ways by professionals, while the discussions were dominated by a few panelists. Personal views could interfere with our intentions to objectively evaluate the existing data. To help with the transparency of the decision process, personal views should be made as clear as possible [3]. Therefore, we surveyed our panelists to analyze their views and opinions and to explore the reasons for the differences in the controversial area s safe upper target phenylalanine levels in adults with PKU.

Explanatory box: phenylketonuria Phenylketonuria (PKU) is an autosomal recessive disease caused by an inborn error of phenylalanine metabolism. The incidence in Europe is estimated to be 1:10.000 newborn [6]. There is a deficiency in the enzyme phenylalanine hydroxylase that converts phenylalanine into tyrosine. In untreated PKU, elevated phenylalanine levels in blood and brain cause severe intellectual disability, epilepsy, and behavioural problems.

Due to newborn screening programs, established around the 1960–1970s, PKU can be detected and treated early in life, substantially improving outcomes. For most patients, a burdensome natural protein restricted diet accompanied by amino acid supplements is the main form of treatment. Alternative treatments are (1) a synthetic form of the co-enzyme tetrahydrobiopterin (sapropterin), however only a subset of patients are fully responsive and (2) enzyme substitution therapy, however this is not widely available in Europe (pegvaliase).

In 2017, the first European PKU guidelines for diagnosis and treatment of PKU was published [7, 8]. In 2018, a new panel of professionals started to work on the recently published revision [5]. One of the most difficult statements to reach consensus on was treatment targets in adult PKU (excluding pregnancy). The long-term effect of increased phenylalanine levels is not yet clear, as this is the first cohort of early-treated adults reaching middle-age. Available data document sub-optimal outcomes in a variety of areas, such as cognition, social functioning, mental health, brain health (neuroimaging, and brain metabolism), heart and kidney functioning, vision (eye movements) and oxidative stress (biomarkers). However, the importance of metabolic control in adulthood remains uncertain given the intercorrelation between levels of metabolic control at different ages, which may all contribute to outcomes in adulthood. On the other hand a diet for life is associated with a significant time and financial burden (i.e. meal planning) [9] and a psychosocial burden (i.e. feelings of exclusion, stress and guilt) [10], which may impact mental health. Thus, clinicians find it difficult to arrive at decisions which balance benefits and burden of treatment. Internationally, opinions for treatment targets vary from < 360 to < 900 µmol/l or even higher [11, 12].

## Methods

A web-based questionnaire was developed and circulated, following group plenary meetings in which current literature regarding adult treatment targets was discussed.

The survey link was sent to all members of the advisory board (including the guideline lead: FvS and coordinator: AvW; n = 23) in the fall of 2022. Qualtrics© (https://www.qualtrics.com/) was used to record the questionnaire responses. Technical functionality was tested beforehand. All results were collected in an anonymous way.

The questionnaire examined four areas: (1) The demographics of the professionals involved in the survey (including five questions); (2) Specific opinions on the recommendations of phenylalanine levels in adulthood (three questions); (3) Interpretation of scientific data (two questions); and (4) General views on the development of these adult-specific treatment guidelines (a sub-questionnaire with 17 items).

The questions on target blood levels were based on the European PKU guidelines published in 2017 and on the American PKU guidelines published in 2014 [7, 8, 11]. Interpretation of scientific data was probed by asking our panelist which outcomes they defined critical, important, or less important in evaluating the outcomes of adult treatment. Moreover, we asked them what degree of deviation from a normal distribution (mean standard deviation), they would consider clinically relevant when judging neuropsychological functioning. The questions probing views regarding the importance of staying on diet in adulthood stemmed from group discussions during meetings of the guideline panel. These questions (Table 5) directly reflected comments and arguments that were given during the group discussions. The wording of these options was often suggestive, exemplifying how individuals might attempt to influence discussions through choice of words rather than rational arguments or hard evidence. By including these options in the survey, our aim was to quantify these feeling-based opinions. Please note that although some questions may seem technical, all panelists were experienced in reading the literature and performing research in PKU and all were provided with the same literature. Therefore, they represent an ideal group to examine differences in opinion in the face of similar exposure to research findings.

Data were collected between June and November 2022 and analyzed with SPSS version 23 (IBM). Statistical analyses were carried out using one-sample t-test when appropriate.

Ethical consent was not sought as no personal data has been collected.

## Results

### Demographics of respondents

Nineteen of the 23 participants (83%) completed the survey. Their characteristics are displayed in Table 1.

**Table 1:** Characteristics of the professionals (questions 1–5)

	Number
*Professionality*	
Paediatric specialists*	11
Adult specialist	1
Dietitian / nutritionist	4
Research neuropsychologist	2
Biochemist	1
*Treating population*	
Children only	1
Adolescents only	1
Children and adolescents only	4
Adults only	1
Children, adolescents, adults*	9
I do not treat patients	3
*PKU patients < 18 years in center*	
1–50	1
51–100	7
101–200	5
201–300	1
> 300	1
Not applicable	4
*PKU adult patients in center*	
1–50	4
51–100	6
101–200	3
201–300	0
> 300	1
Not applicable	5
Experience in treating patients (years)**	Median: 28.5 Range: 13–45 years

*7 Paediatric specialists and 2 dietitians/nutritionist are treating children, adolescents and adults

**This question was filled out by n = 16

### Target blood phenylalanine levels

**Fig. 1:**
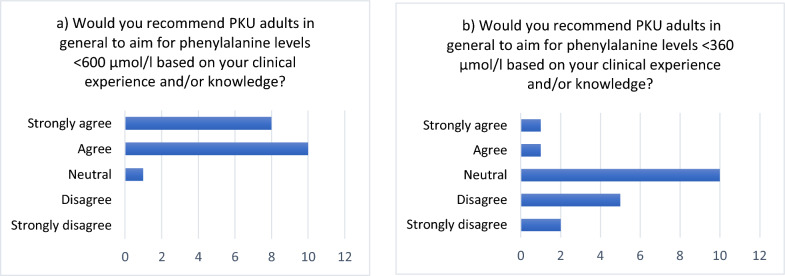
Questions 6 and 7 regarding target phenylalanine levels in adults. Number of respondents providing different degree of agreement

Most of the respondents (n = 18; 95%) agreed to recommend that, generally, PKU adults should aim to maintain phenylalanine levels below 600 µmol/l (Fig. 1a). Two respondents (11%) agreed to recommend maintaining phenylalanine levels < 360 µmol/l (Fig. 1b). Concerning the different types of risks that participants considered acceptable, two-thirds of the participants (n = 13) would prefer to be on the safe side and recommend phenylalanine levels stricter than needed rather than run the risk of cognitive impairments. In other words, they preferred overtreating rather than undertreating patients. Four participants preferred a more lenient approach, potentially undertreating patients (see Table 2).

**Table 2:** Question 8. Number (%) of respondents (out of 19) accepting different kinds of risk in answering the question: *Currently, there is no consensus about the influence of concurrent phenylalanine or phenylalanine during various life phases on neuropsychological functioning and mental health during young and late adulthood. Based on your knowledge and experience, would you rather be on the safe side and aim for strict phenylalanine control (potentially overtreating) or would you rather suggest a more lenient approach as the treatment can be burdensome and may have side effects (potentially undertreating)?*

Statement	Number (%) of respondents agreeing
I accept the potential risk of overtreatment	13 (68.4%)
Neutral	2 (10.5%)
I accept the risk of potential undertreatment	4 (21.0%)

Based on your knowledge and experience, would you rather be on the safe side and aim for strict phenylalanine control (potentially overtreating) or would you rather suggest a more lenient approach as the treatment can be burdensome and may have side effects (potentially undertreating)?

### Interpretation of scientific data

Participants were presented with different options regarding the importance of outcomes in different domains for people with PKU. Even though neurocognitive outcomes are the most prominent outcome in PKU, neurological and mental health disorders are also reported and a possible involvement of hearth and/or kidney (mainly in adults) has been suggested in the literature.

The great majority of participants considered cognitive, behavioural and well-being outcomes to be critical in judging the success of therapy in people with PKU with rates ranging from 63.2–94.7%; neurophysiological, biological and other markers of organ health were considered less critical with rates ranging from 5.3–26.3% (Table 3).

**Table 3:** Number (%) of respondents (out of 19) answering the question: *Which health benefits and harms of a therapy are important to the decision regarding the optimal management strategy in adults?*

	Critical	Important, but not critical	Of limited importance
Cognition (IQ)	12 (63.2%)	6 (31.6%)	1 (5.3%)
Executive functions (i.e. attention, working memory)	16 (84.2%)	4 (21.1%)	0
Mental health (i.e. mood, depression, anxiety, ADHD)	18 (94.7%)	1 (5.3%)	0
Neurological functioning (i.e. tremor, neurological deterioration)	16 (84.2%)	1 (5.3%)	2 (10.5%)
Social functioning (i.e. education/job, relationship)	16 (84.2%)	3 (15.8%)	0
Quality of life / psychosocial functioning	17 (89.5%)	2 (10.5%)	0
Neuro Imaging / white matter abnormalities (MRI)	3 (15.8%)	10 (52.6%)	6 (31.6%)
Brain metabolism (MRS / PET)	2 (10.5%)	13 (68.4%)	4 (21.1%)
Neuro activity (EEG / VEP)	2 (10.5%)	8 (42.1%)	9 (47.4%)
Oxidative stress	1 (5.3%)	7 (36.8%)	11 (57.9%)
Other organs: heart functioning	5 (26.3%)	6 (31.6%)	8 (42.1%)
Other organs: kidney functioning	4 (21.1%)	9 (47.4%)	5 (26.3%)
Other organs: eye functioning*	5 (26.3%)	7 (36.8%)	6 (31.6%)
Other organs: bone health	2 (10.5%)	11 (57.9%)	6 (31.6%)
Other organs: gut health	2 (10.5%)	11 (57.9%)	6 (31.6%)

*This outcome was not answered by 1 participant (5.3%)

Interpretation of scientific data was also probed by asking our participants which degree of deviation from a normal distribution they would consider to be clinically relevant when interpreting neuropsychological findings. This question was asked because, during the panel discussions, it seemed that people interpreted group z-scores and effect sizes of neurocognitive impairment differently giving them different weight and clinical significance.

Most participants (10/19 = 53%) considered a significant difference of 0.5–1 SD to be relevant. Only few participants (3/19 = 16%) considered relevant only a larger difference, while 5 participants (26%) did not find any of the options to be clinically relevant (Table 4).

**Table 4:** Number (%) of respondents (out of 19) agreeing to statements in answer to the question: *When would you interpret a neuropsychological finding in an article as being of clinical relevance?*

Statement	Number (%) of respondents agreeing
PKU mean < 0.5 standard deviation from the normal range/population but still statistically significant	1 (5.3%)
PKU mean > 0.5 standard deviation from the normal range/population	4 (21.1%)
PKU mean > 1 standard deviation from the normal range/population	6 (31.6%)
PKU mean > 1.5 standard deviation from the normal range/population	1 (5.3%)
PKU mean > 2 standard deviation from the normal range/population	2 (10.5%)
None of the above	5 (26.3%)

### General views about treatment

There were two statements where all participants agreed, namely that higher levels of phenylalanine during early and middle-aged adulthood may increase risks in older age and that dietary treatment is a limiting factor for the social life for at least some patients (Table 5). For six statements agreement was more > 80% (questions 1, 5, 8, 9, 10, 17) of which three addressed the impact of high blood phenylalanine levels in adulthood (questions 5, 9, 10).

**Table 5:** Ratings of different statements preceded by the following: *Below you can find views of professionals in the guideline development group towards strict or less strict treatment targets during adulthood, based on clinical experiences*. *Please select your level of agreement per item.* Scores in the tables are ratings by the 19 participants, thus levels of agreement could vary between 19 and 0. For questions wiht an alterisk disagreement is consistent with views that it is important to maintain treatment. For all other questions is the other way round

Statement	Strongly agree	Agree	Neutral	Disagree	Strongly disagree
*History of PKU science*					
1. The history of PKU is full of examples of previously incorrect thoughts regarding the age when stopping treatment is safe or regarding the upper phenylalanine level that is safe, so let’s not make the same mistakes in adult treatment	10	6	3	0	0
2. The history of medical science shows that many decisions based on presumably ‘solid data’ proved wrong later on, so, even in rare diseases such as PKU only the strongest results from RCT must be considered*	1	7	3	7	1
*Life phases*					
3. Since we know that phenylalanine during childhood and adolescence can affect outcomes later in life, higher phenylalanine during early and middle-aged adulthood may increase risks in older age	10	9	0	0	0
4. Childhood and old age are the most vulnerable periods for the brain health. Similarly, the ages when phenylalanine may be most damaging for the brain could be childhood and old age with early and middle-aged adult brains being less vulnerable	5	6	6	2	0
*Adulthood*					
5. Case reports of previously undiagnosed PKU patients with neurological symptoms which present only in adulthood and resolve after treatment initiation show that high phenylalanine during adulthood affects outcome	8	10	0	1	0
6. Case reports of previously undiagnosed PKU patients with neurological symptoms which present only in adulthood and resolve after treatment initiation show that treatment is only needed when clinical symptoms appear*	1	4	0	9	5
7. Many adult PKU patients live happy and fulfilled lives without strict metabolic control, implying less/no vulnerability of phenylalanine levels in adulthood*	1	5	6	6	1
8. Patients themselves often do not see that they have issues, while these are evident to others (i.e. family members)	7	11	1	0	0
9. Patients who decrease phenylalanine with Palynziq report getting out of ‘brain fog’ when phenylalanine decreases to normal levels. This shows that high phenylalanine levels influence brain functions even in adulthood	7	10	2	0	0
10. Adult PKU patients with high phenylalanine levels can suffer from anxiety and headaches. This shows the influence of high blood phenylalanine even in adulthood	7	10	2	0	0
11. The anxiety and headaches of adult PKU patients are due to their feeling of guilt at being unable to keep lower phenylalanine levels rather than to higher phenylalanine levels themselves*	2	2	8	6	1
12. Adult PKU patients can be somewhat socially awkward. This is related to high phenylalanine levels during adulthood	2	8	8	1 lePara>	0
13. Adult PKU patients can be somewhat socially awkward. This may be due to the rigid diet they followed in childhood	1	9	7	1	1
14. Dietary treatment is a limiting factor for the social life for at least some patients*	8	11	0	0	0
15. Partners of PKU women wish that they would always stay pregnant since pregnancy makes them easier to deal with. This shows that higher adult phenylalanine levels do have an impact on mood and behaviour	8	6	3	2	0
16. PKU women generally return to a higher phenylalanine diet after giving birth, implying they do not experience better daily functioning at lower phenylalanine levels*	1	2	3	10	3
17. PKU women relaxing the diet after giving birth does not imply that there is no effect of high phenylalanine, but that the diet is too burdensome for them	9	7	2	1	0

Sixteen of our questions explored views on treatment necessity (question 13 was not relevant). To evaluate the degree of consensus on the importance of maintaining treatment in adulthood, we have scored the answers consistent with this view (+ 2 for strongly agree, + 1 for agree) and contrasted them with the answers indicating disagreement (− 2 for strongly disagree, − 1 for disagree; reversing scores for agreeing/disagreeing as appropriate). The minimum and maximum scores possible were − 32 and + 32, respectively. The average score per participant was 11.7 (range 3 to 25), which was statistically different from a null effect of 0 (one sample t-test; t(8) = 9.61, *P* < 0.001), indicting a general preference for treatment.

## Discussion

Our study surveyed the personal opinions and views of professionals that were involved in the development of the recently published first revision of the European PKU guidelines [5]. Developing recommendations was based foremost on reviewing and evaluating available scientific evidence. However, especially formulating a recommendation on the therapeutic target blood Phe levels for adults involved an intense debate. The available literature was presented and discussed during plenary meetings. Participants stood at opposing ends supporting either levels equal or less of current recommendation (< 600 µmol/l) or argued for having no target level at all [7, 8]. Panel members who argued for a target Phe recommendation of < 600 µmol/l cited clinical trials with a low number of participants and cross-sectional data showing impairments in groups with higher levels [13–16]. Panel members who argued against the use of a ‘one-size-fits-all target level’, stressed historic metabolic control being more responsible for impairments than current adult levels, the psycho-social burden of the diet, and the strong individual variability in outcomes in adults with PKU [17].

Through this study, we sought to explore possible reasons for the differences of opinion within this relatively small panel of experts who gathered together to formulate guidelines for the treatment of PKU. We aimed to quantify how opinion may differ even among professionals with strong expertise in research and clinical care and after a thorough discussion of the same literature.

Before addressing the results of the study, we want to point out two important limitations. The most important was that, unfortunately, not all the panel members participated in the survey, although we achieved a participation of over 80%. The panel members that did not participate strongly argued for more personalized target levels because they felt that the current upper target of 600 µmol/l would be unnecessarily strict for many patients. In fact, three of the four panel members that did not participate left the guideline group because they could not support the recommendation that had reached consensus of > 75%. It is also important to note that the panel included only one specialist for adults with PKU (even though nine members treated all age groups) and no patient representatives (Table 1), which might have influenced the panel discussion and the study results. The second limitation is that, since our study has no precedent, our questions should be considered a first attempt that should be improved in further studies. For example, we could have incorporated specific sections of research articles in the survey to ask more detailed questions regarding data interpretation. In addition, differences in interpretations could have been better appreciated by following our questions about effect sizes and other topics where strong opinions or feelings were expressed, with open questions which could have then been analyzed qualitatively.

Our results will be discussed in two sections.

### Interpreting scientific evidence

It is important to clarify what clinicians consider crucial outcomes in judging treatment success. This will help to resolve or clarify disagreements, as is suggested by GRADE [4]. In our survey, most participants agreed that cognitive outcomes are critical, but IQ was rated less critical than executive functions. This can be explained as IQ seems to be mainly influenced by phenylalanine during childhood and adolescence [18, 19]. They also agreed that outcomes related to everyday functioning and well-being are important, but they put less weight on neurophysiological and biological markers. However, there was variation. Some professionals considered (affected) brain metabolism an important outcome. This shows how professionals may use different results in informing their opinion regarding the necessity of treatment targets.

Our survey also addressed the size of differences from controls which should be considered relevant when deciding on the balance of benefits and harm for an intervention (e.g., dietary treatment). A preliminary consideration is that with enough participants, even a tiny difference in reaction times between groups (measured in milliseconds) may be statistically significant but it may have no clinical relevance. What is more important than statistical significance, is the *size* of the difference which can be measured in units of standard deviations. A second consideration is how differences from controls relate to quality of life. In PKU, one can ask whether faster performance on a neurocognitive task or an improvement in the blood brain barrier transport of tyrosine, shown with positron emission tomography, are clinically relevant markers of therapy success [20–22]. Answers varied, although most of our panelists answered that a significant difference of 1 SD or even less in a neuropsychological task should be considered clinically relevant, while 16% of participants (but note small n = 3) considered relevant only larger differences > 1.5 SD. These different answers reflect the group discussion. Of course, interpretation of effect sizes depends on many aspects, such as type of outcome, study duration, and patient numbers. However, *group* SD difference and *individual* SD difference were both used in literature and during the discussions, even though they have a different interpretation. In neuropsychological testing, only *individual* score < 2 SD from the reference group are clearly outside the normal range. So, some panelists focused on the data regarding (individual) neuropsychology results < 2 SD. Even though even small speed differences are likely to be clinically relevant and can cumulate in complex everyday tasks creating a disadvantage compared to peers (e.g., in writing an essay, food shopping, organizing a birthday party, organizing finances, etc.). Some panelists focused on group results. When considering group results, much smaller differences are relevant. Average scores hide individual variability. If the PKU scores are normally distributed but shifted of 1 SD from the control distributions, this means that 15.9% of the PKU participants will have scored of 2 SD from the controls compared to only 2.3% of the control participants. These differences would be higher if the PKU distribution is left skewed with more values on the impaired size of the distribution. Whether and how individual variability in the PKU populations is acknowledged in interpreting research results should be explored in further studies.

### General views about treatment and decision making

Most of the respondents in our survey (n = 18; 95%) would recommend that PKU adults should, in general, aim to contain Phe levels below 600 µmol/l (in line with the first and updated European guideline on PKU [5, 8] while two respondents (11%) favoured Phe levels < 360 µmol/l (in line with the current USA guideline on PKU [11]. As previously mentioned, the panelists debated whether to set a treatment target of < 600 µmol/l or to have no treatment target at all. One of the respondents remarked in a note to personally accept phenylalanine levels up to 900 µmol/l in clinical practice when the patient is doing fine. This remark is in line with the commentary of Burgard et al. on the first European guideline [8, 12]. It well illustrates the differences between guidelines and clinical practice. Guidelines apply to all PKU patients in general, however, in practice, guidelines should be adapted to the specific circumstances of individual patients with appropriate deviations. Another respondent remarked to have witnessed remarkable benefit from lowering phenylalanine to physiological levels in some patients, which he/she considered achievable only with pharmacological treatments. It is unclear how much these opinions are based on (unpublished) but systematically collected data or on unsystematic personal observations which can be affected by recall bias [3].

The benefits of an intervention should always be considered against potential harm. Even for clinically relevant improvements, one should ask if they outweigh the burden of a demanding treatment, which, in the case of PKU, may be an especially burdensome diet. Therefore, an important consideration in reaching treatment recommendations is the willingness to risk undertreating versus overtreating patients. In our survey, all the respondents acknowledged that dietary treatment is a limiting factor for social life (Table 4, question 14; n = 18 100%). Still, most (n = 12; 67%) preferred the risk of overtreating. Only four respondents (22%) preferred undertreatment and two respondents (11%) chose neither. It is likely that the preference of our participants for overtreating was linked to the acknowledged possibility that higher phenylalanine levels during early and middle-aged adulthood could increase risks of cognitive impairments in older age (Table 4 question 3; n = 18 100%). Other studies have reported that, in the absence of clear data, guideline members prefer intervention over non-intervention [23].

Since all respondents acknowledged the social burden of the diet and at the same time the potential negative impact of high phenylalanine levels later in life, differences in treatment preferences are likely linked to preferences in clinical decision making. Professionals can be described either as interventionist (disease-oriented) or health maintenance-oriented (or patient-oriented). The first tends towards immediate action, whereas the second are willing to wait and observe the evolution of the disease [24]. Also, experience and emotional state of mind can play a role [24, 25]. One of the respondents remarked that we, as professionals, have made mistakes in the past when choosing treatment pathways, and we should be cautious because, once a treatment is stopped, it is very difficult to re-establish. On the other hand, another respondent stated their preference to accept risks of undertreatment for adult patients if this avoids alienation and allows acceptance of at least some treatment. These personal experiences once more demonstrate that opinions are not based solely on empirical evidence.

Despite individual differences, most of our respondents expressed views consistent with the importance of maintaining (dietary) treatment in adulthood. Guidelines have the purpose of providing general advice, but clearly this should be individually-tailored following consultation between the health professionals and the patient involved. The importance of an individual approach was stressed by panelists in group discussions and in comments to the questionnaire.

## Conclusion

To summarize, our survey described some examples of the personal views of panelists participating in the development of the recently published second edition of the European PKU guideline, that could play a role in the judgements experts make on reaching statements in guideline. By doing so, it may help to explain the differences in interpreting the evidence and formulating a treatment target for adults with PKU. Most panelists responded to the survey. Regarding the evidence interpretation it was suggested that some panelists considered different outcomes as critical and that effect sizes of neuropsychological test results were differently interpreted. A first step would be to discuss such differences to be able to agree on the available literature interpretation, even if it is ambiguous. Most panelist in our survey expressed a preference for being on the safe side when choosing a treatment target, thus potentially overtreating rather than undertreating patients. This aligned with the number of panelists who preferred a treatment target of < 600 µmol/l versus no general treatment target. However, these panelists also acknowledged the burden of treatment and the difficulty of maintaining it lifelong, with possible impacts on mental health. Critically appraising evidence remains at the core of informed clinical decisions. Recommendations and group discussions are valuable to this end. However, our survey showed that individual preferences and professional experiences vary and it is likely that they will influence opinions more when strong, relevant evidence is lacking. In this case, relying on personal opinions is unavoidable and not necessarily negative. A Delphi anonymous survey is a possible tool to allow opinions to be freely expressed and collated, especially when discussions are dominated by only a few. At the same time, making professionals more aware of their own personal biases and preferences will make the process of reaching decisions clearer and more transparent, and reduce the risk of emotions influencing the content of the recommendations. The results can be used to discuss more easily within the group. Our study is a preliminary attempt in this direction. However, even more importantly, future research should consider what are the values and preferences of patients with PKU in balancing the desirable effects of treatment with negative effects.

## Data Availability

All data generated or analysed during this study are included in this published article.
